# Recapitulating cortical development with organoid culture *in vitro* and modeling abnormal spindle-like (ASPM related primary) microcephaly disease

**DOI:** 10.1007/s13238-017-0479-2

**Published:** 2017-10-23

**Authors:** Rui Li, Le Sun, Ai Fang, Peng Li, Qian Wu, Xiaoqun Wang

**Affiliations:** 1 State Key Laboratory of Brain and Cognitive Science, CAS Center for Excellence in Brain Science and Intelligence Technology (Shanghai), Institute of Biophysics, Chinese Academy of Sciences, Beijing, 100101 China; 2 University of Chinese Academy of Sciences, Beijing, 100049 China; 3 Beijing Institute for Brain Disorders, Beijing, 100069 China

**Keywords:** neocortical development, cerebral organoid, microcephaly, ASPM

## Abstract

The development of a cerebral organoid culture *in vitro* offers an opportunity to generate human brain-like organs to investigate mechanisms of human disease that are specific to the neurogenesis of radial glial (RG) and outer radial glial (oRG) cells in the ventricular zone (VZ) and subventricular zone (SVZ) of the developing neocortex. Modeling neuronal progenitors and the organization that produces mature subcortical neuron subtypes during early stages of development is essential for studying human brain developmental diseases. Several previous efforts have shown to grow neural organoid in culture dishes successfully, however we demonstrate a new paradigm that recapitulates neocortical development process with VZ, OSVZ formation and the lamination organization of cortical layer structure. In addition, using patient-specific induced pluripotent stem cells (iPSCs) with dysfunction of the *Aspm* gene from a primary microcephaly patient, we demonstrate neurogenesis defects result in defective neuronal activity in patient organoids, suggesting a new strategy to study human developmental diseases in central nerve system.

**Electronic supplementary material:**

The online version of this article (doi:10.1007/s13238-017-0479-2) contains supplementary material, which is available to authorized users.

## Introduction

The most highly evolved structure in the human brain is the neocortex, which is responsible for the higher cognitive functions unique to humans (Rakic, 2009; Borrell and Reillo, 2012). The dramatic human neocortical expansion and gyri formation underline the precise orchestration of neurogenesis and neuronal migration in the embryonic stage, especially during the peak of neurogenesis, which occurs during the second trimester of gestation in humans (Kriegstein et al., 2006; Lui et al., 2011; de Graaf-Peters and Hadders-Algra, 2006). Radial glial (RG) cells constitute a major population of neural progenitor cells and are referred to vRG cells (ventricular radial glial cells); these cells occupy the proliferative ventricular zone (VZ) (Noctor et al., 2002; Hartfuss et al., 2001; Anthony et al., 2004). vRG cells display interkinetic nuclear migration (INM) behavior, proliferate extensively at the luminal surface of the VZ and predominantly undergo asymmetric division to self-renew while simultaneously giving rise either directly to neurons or to an IPC that subsequently divides symmetrically to produce neurons (Kriegstein et al., 2006; Noctor et al., 2001; Fishell and Kriegstein, 2003; Huttner and Kosodo, 2005). A new population of neural stem/progenitor cells has recently been reported to occupy the greatly expanded subventricular zone (SVZ), which is composed of intermediate progenitor cells (IPCs) and outer radial glial (oRG) cells and can be divided into the inner (ISVZ) and outer SVZ (OSVZ) by the inner fiber layer (IFL) in primates (Dehay and Kennedy, 2007; Hansen et al., 2010; LaMonica et al., 2012; Garcia-Moreno et al., 2012). Unlike vRG cells, oRG cells show distinctive mitotic somal translocation (MST) behavior, giving rise to IPCs in humans (Hansen et al., 2010; LaMonica et al., 2012; Ostrem et al., 2014; Taverna and Huttner, 2010; Gertz et al., 2014) instead of neurons, as in mice (Wang et al., 2011), and serve as transit amplifying machinery for proliferative pool expansion (Lui et al., 2011; Fietz and Huttner, 2011; Pontious et al., 2008). An enlarged OSVZ, accompanied by an increase in both oRG cells and IPCs, may account for neuronal amplification and the tangential dispersion of neurons that contributes to cortical expansion and gyrification (Borrell and Reillo, 2012; LaMonica et al., 2012; Reillo et al., 2011; Nonaka-Kinoshita et al., 2013).

Extensive studies have demonstrated that the neocortex is a six-layered laminated structure generated by a specific pattern of neuronal production and migration. Newborn neurons migrate successively following the guidance of radial glial fibers over the existing early-born neurons during cortical development and occupy superficial layers in the cortical plate (CP), creating cortical layers (CL). Cortical lamination provides the basic framework for neocortical function (Gao et al., 2013; Douglas and Martin, 2004). Recent achievements suggest that the developing human neocortex differs significantly from the rodent model; however, due to limitations on the experimental manipulation of human tissues and on the monitoring of long-term development in patients, much of the recent progress in understanding cortical development has come from rodent studies (Wang et al., 2011; Caviness and Rakic, 1978; D’Arcangelo, 2006). Therefore, new research strategies need to be established to study human cortical development.

Several previous efforts to grow neural tissues in culture dishes, such as an optic cup (Eiraku et al., 2011), a pituitary gland (Suga et al., 2011), cortex-like cell layers (Nasu et al., 2012; Kadoshima et al., 2013; Eiraku et al., 2008), some brain-region-specific organoids (Qian et al., 2016; Pasca et al., 2015), entire brain-like organoids (Lancaster et al., 2013; Camp et al., 2015), and even organoids with surface folding (Li et al., 2017) have been reported. Also, organoids have been proven to be a remarkable model to study diseases, such as ZIKA virus (Qian et al., 2016; Cugola et al., 2016; Garcez et al., 2016; Nowakowski et al., 2016; Tang et al., 2016; Xu et al., 2016) and neural system disease (Lancaster et al., 2013; Bershteyn and, 2017). Therefore, organoid culture system is suitable for modeling neurodevelopmental diseases and related cognition defects.

To address this issue, we have developed a new three-dimensional culture system for a cortex-like tissue derived from induced pluripotent stem cells (iPSCs) *in vitro*. Here, we show that these cultured organoids recapitulate the lamination organization of the layered neocortex generated from vRG, oRG, and IP cells and eventually mimic the cortical development *in vivo*. Finally, in an investigation of the ASPM-dependent pathogenesis of microcephaly, we report that the cortical organoids generated from primary microcephaly patient-derived iPSCs were unable to form normal cortical lamination resulting in defective neuronal activity.

## Results

### Self-organized organoids mimics early cerebral development

**Figure 1:**
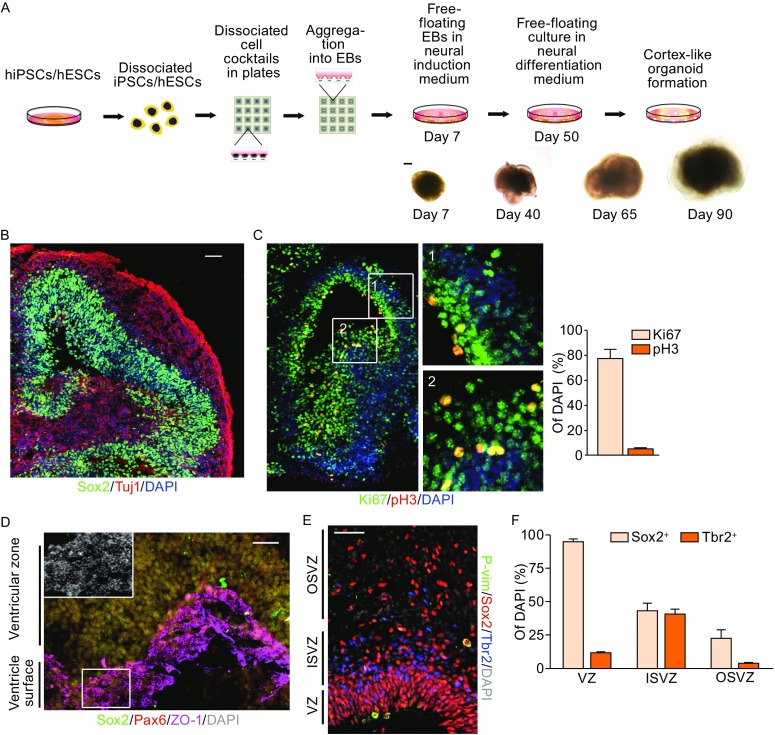
**The procedure for** ***in vitro*** **generation of a human cortex-like organoid**. (A) Schematic diagram of the culture system for cortex-like organ formation *in vitro*. Representative bright field (BF) images of each stage. (B) Immunostaining results for the progenitor marker (Sox2, green) and new-born neuron marker (Tuj1, red) organoids on day 36. (C) Immunostaining for proliferating cells: Ki67 (green), pH3 (red, mitotic marker). The percentage of Ki67^+^ and pH3^+^ cells was shown on the right. (D) Immunostaining image for cortical NE-like structure after 36 days of neural induction. Sox2 (green), Pax6 (red), ZO-1 (magenta), and DAPI (grey). (E) Staining for Tbr2 (blue) and Sox2 (red) revealing OSVZ localization of basal progenitors. (F) Quantification of Sox2^+^ or Tbr2^+^ percentages within all progenitors in the VZ, ISVZ and OSVZ, respectively. All data are presented as means ± s.e.m. Scale bars: 200 μm in (A), 50 μm in (B–E)

To develop a paradigm generating functional cerebral organoids, we used free floating embryonic bodies (EBs). Approximately 3 × 10^6^ dissociated hiPSCs or hESCs were plated onto a low cell-adhesion plate and uniformly sized tight embryonic body-like aggregates formed within the first 7 days (Fig. 1A). On day 7, the aggregates were transferred to petri dishes for neural induction culture. The resulting 3D aggregates were then re-plated onto petri dishes for neural differentiation on day 50. The optimized culture conditions allowed these cell aggregates to continue to differentiate and mature beyond 120 days (Fig. 1A). To understand whether neurogenesis in organoids follows *in-vivo* development pattern, we dissected organoids at early stage. On day 36, the hiPSC-derived organoid grew fast and formed a ventricle-like cavity surrounded by neural progenitors (Fig. 1B). Around the ventricle, a thick layer of Sox2-positive progenitor cells and some expanded Tuj1-positive new-born neurons were observed in organoids, indicating neural progenitors and new-born neurons generated in these organoids (Fig. 1B). Also, the proliferative marker Ki67 and phospho-histone H3 (pH3), a marker for the G_2_-M phase, were detected in progenitor cells close to the ventricle. Around 77.21% cells were Ki67 staining positive in ventricular area, suggesting that early development of cortical development primarily focuses on cell proliferation (Fig. 1C). To further characterize the dorsal telencephalic progenitor cells, we stained ZO-1, Pax6, and Sox2 (Fig. 1D). The progenitors in the inner area exhibited ring structures of tight junction reviewed by ZO-1 (Fig. 1D), resembling a lateral ventricle with characteristic apical localization of the ventricular radial glia (vRG) (Gotz and Huttner 2005; Pilz et al., 2013).

To examine RG identity during the early development of these cerebral-like 3D tissues, we performed staining for phospho-vimentin (P-vim) and Sox2 on day 36 (Fig. S1A). We observed vRG for Sox2^+^ cells along the apical side with apical anchor and extended basal cellular processes toward the outer surface of these tissues in cerebral organoids. Since vRG cells typically divided on the lumen surface, we observed a series of mitotic cells at different dividing stages in organoids, such as prophase, metaphase, anaphase and telophase (Fig. S1A), resembling innate vRGs features in early human neocortical development. In primates, the population of oRGs is significantly larger than in rodents, and these oRGs play critical roles in neocortical neurogenesis. Sox2^+^/P-vim^+^ cells above VZ region exhibited long basal processes (Fig. S1A), suggesting that oRGs also exist in organoids. In consistent with previous proliferating marker staining results, we observed 19.55% and 6.39% mitotic vRGs and oRGs in VZ/SVZ area respectively (Fig. S1A).

In mammals, vRG cells initially divide symmetrically to enlarge the progenitor pool and then divide asymmetrically to maintain one daughter cell as vRG and the other as a progenitor cell, such as an oRG cell, an IP cell, or a newborn neuron (Hansen et al., 2010; Wang et al., 2011); hence the cell division behavior is a determinant for neural progenitor pool and cortical neurogenesis. To confirm division behavior of vRGs in cerebral-like tissue, we assessed the angle between cleavage plane and ventricle surface (Fig. S1B). In our hiPSC culture, proliferating apical progenitors preferentially divided from a “vertical” cleavage plane (60–90°, 57.0%; Fig. S1C) on day 36, indicating that vRGs primarily undergo symmetrically division to enrich vRG pool and also contribute to progenitor or neuron production at early culture time, which is similar to early cortical development. Additionally, we use time lapse to observe the activity of these vRG cells, and found typically interkinetic nucleus movement (INM) like vRG *in vivo* (Fig. S1D and Movie S1). These results indicate vRGs in organoids mimicked cellular activity *in vivo*.

To determine whether features of human brain development with oRG and IP cell existence were recapitulated in cerebral organoids, we analyzed the organoids on day 65 (Fig. 1E). The distribution of Sox2^+^ progenitors were examined and we observed a population concentrated close to the apical surface (VZ) that was thought to be vRG cells, as well as another population displaced from the apical surface (OSVZ), that was consistent with oRG identity in organoids (Fig. 1E and 1F). In addition, IP cells identified by Tbr2 staining primarily localized outside of but close to the VZ on day 65 (Fig. 1E and 1F). The presence and distribution of RGs and IP cells demonstrated that this new 3D culture system could mimic early development of brain with all types of known neural progenitors in dish.

### Spatial neuron lamination of organoids

**Figure 2:**
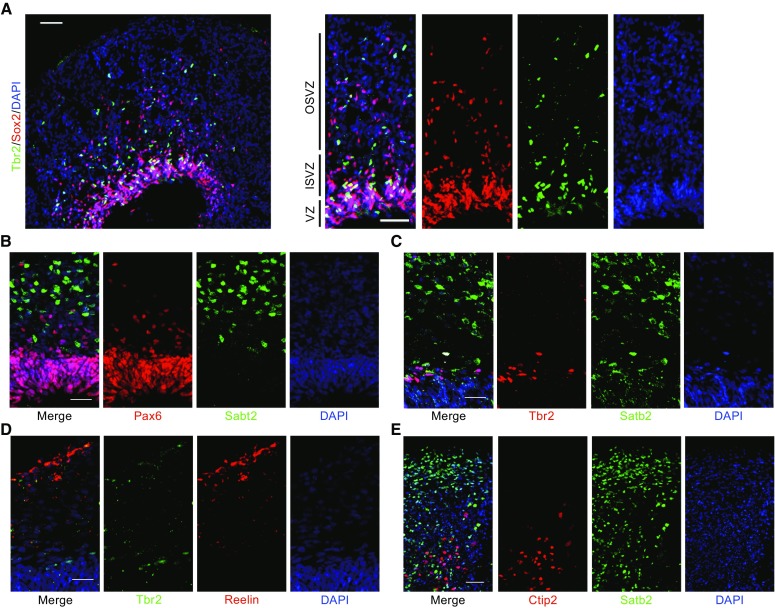
**Cortical spatial lamination in 3D organoid culture**. (A) Cryosections of organoids were immunostained for progenitor markers on day 65. (B–E) Immunostaining results of progenitor and pyramidal markers in organoids on day 65 (B–D) and day 90 (E). Scale bars: 50 μm in (A–E)

To assess neuron production abilities, neural progenitor cells were analyzed in the organoids on day 65 (Fig. 2A). The abundance and distribution of Sox2^+^ vRGs and oRGs, as well as Tbr2^+^ IPCs, were remarkably similar to human neocortex cytoarchitecture at GW16 (Fig. S2A), suggesting that this method could reproduce the fine structure of cerebral cortex, such as OSVZ (Fig. 2A).

The mammalian cortical plate consists of six distinct layers. To determine whether organoids could recapitulate the cortical spatial lamination, we stained for cortical layer markers on day 65. In hiPSC-derived neocortex-like tissues, Tbr2 positive subventricular zone (SVZ) was adjacent to the ventricular zone (VZ), which indicated by Sox2^+^ region, this result was consist with structures *in vivo* (Figs. 2A and S2A). Also, cortical plate (CP) indicated by Satb2^+^ neuron located outside of Pax6^+^ and Tbr2^+^ regions (Fig. 2B and 2C). Furthermore, we observed Reelin^+^ neurons along the basal surface (Fig. 2D), suggesting the presence of Cajal-Retzius like cells, which regulates the generation of cortical plate laminar architecture. Finally, in organoid of day 90, we could observe that later-born Satb2^+^ neurons localized more superficially to the early-born Ctip2^+^ neurons (Fig. 2E). This lamination was similar to human developing neocortex (Fig. S2B). Thus, this distribution pattern of dorsal cortical subtype-specific neurons resembles *in vivo* regional sub-specification, indicating these organoids derived from our culture system could efficiently model neocortex organization *in vivo*.

### Electrophysiological properties of neurons in organoids

**Figure 3:**
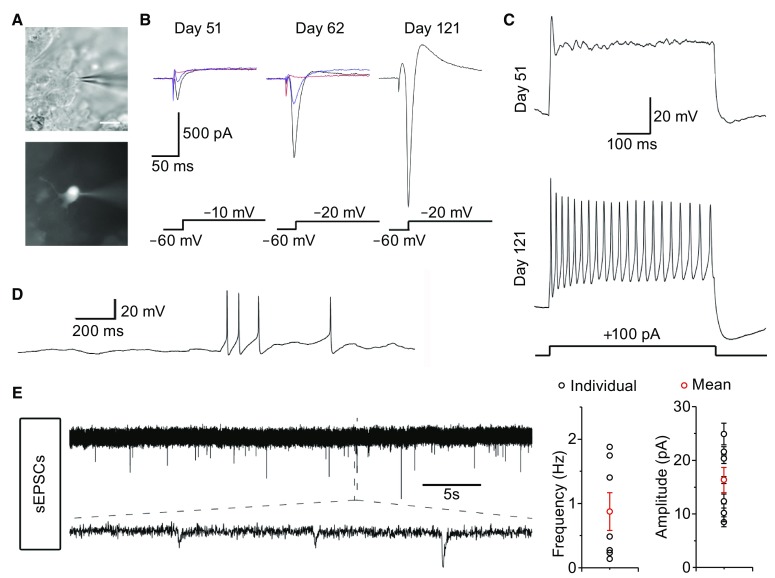
**Physiological properties of cells in organoids at different stages**. (A) An example of cells in organoids recorded under DGC (top) and fluorescence (bottom). (B) Representative traces of membrane currents (black) evoked by depolarizing pulses of −10 mV or −20 mV. The Na^+^ currents were blocked by 1 μmol/L tetrodotoxin (TTX, red) and recovered (blue) after rinsing. (C) Representative traces of membrane potentials in response to a 500-ms current injection from a 51- (top) or 121-day-old (bottom) organoid. (D) Spontaneous action potentials were recorded from a 121-day-old organoid. (E) Spontaneous EPSCs in mature organoids. The average frequency and amplitude are shown on the right. *n* = 7 cells. All data are presented as means ± s.e.m. Scale bar: 20 μm in (A)

With the well-developed lamination in cultured hiPSC-derived organoids, we next investigated the functional characteristics of the neurons using electrophysiology. To examine whether the cells had functional membrane properties, we performed patch-clamp recordings on the outer most layer in cultured organoids (Fig. 3A). In voltage-clamp mode, fast-inactivating inward currents with increasing amplitudes were observed on days 51, 62, and 121 (Fig. 3B). In current-clamp mode, depolarizing the membrane could evoke more action potentials from the cells on day 121 than on day 51 (Fig. 3C). On day 121, the action potential exhibited higher amplitude, lower threshold, and faster kinetic of repolarization than its counterpart on day 51 (Fig. 3C). Spontaneous action potentials could be observed in the cells on day 121 (Fig. 3D). Furthermore, we obtained spontaneous excitatory postsynaptic currents (sEPSCs) from organoids on day 121 (Fig. 3E). These data indicated that the neurons of the organoids underwent a process of maturation; also, chemical excitatory synapses were able to form among cells in the organoids due to the existence of mature like pyramidal neurons at late development stage.

### Organoids recapitulate microcephaly with *Aspm* mutation

**Figure 4:**
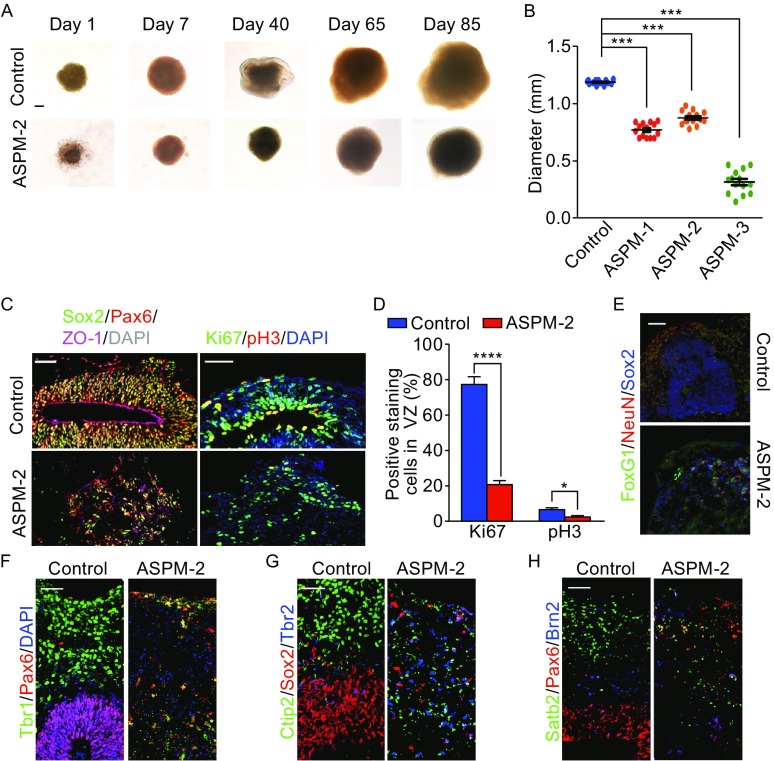
**Human ASPM mutant organoids show disrupted cerebral development**. (A) Representative BF images of control and ASPM-2 organoids at different stages. (B) The diameter of organoids generated from each hiPSC lines. (C) Immunostaining of NE markers in control and ASPM-2 organoids. (D) The percentage of Ki67^+^ and pH3^+^ cells in VZ of the developing organoids. *n* = 3, 3 slices for control and ASPM-2 group. (E) Immunostaining for progenitor markers of control and ASPM-2 organoids on day 65. (F) Immunostaining of telencephalic, neuronal and progenitor marker of control and ASPM-2 organoids on day 65. (G–I) Immunostaining results for progenitor and pyramidal markers of control and ASPM-2 organoids on day 65. All data are presented as means ± s.e.m. *P < 0.05; ***P < 0.001; ****P < 0.0001. Scale bar: 200 μm in (A), 50 μm in (C, E–I)

Human autosomal recessive primary microcephaly (MCPH) is a heterogeneous disorder with mutations in twelve genetic loci (*MCPH1*–*12*) (Faheem et al., 2015; Woods et al., 2005). Mutations in the *Aspm* gene (abnormal spindle-like microcephaly-associated) at the *MCPH5* locus are expected to be the most common cause of human primary microcephaly. The severely reduced brain size observed in patients with *Aspm* mutations has been proven to have difficulty in recapitulating in animal models (Pulvers et al., 2010). To determine whether neocortex-like organization by *in-vitro* 3D culture could be used to mimic neurodevelopmental disorders, iPSCs were generated from reprogrammed skin fibroblasts of a patient with severe microcephaly by retroviral delivery of four well-described reprogramming factors: Oct4, Sox2, c-Myc, and Klf4 (Okita et al., 2007; Takahashi and Yamanaka, 2006). We generated many independent clones and characterized three of these in terms of morphology and pluripotency. All three hiPSC-ASPM cell clones, including hiPSC-ASPM-1, hiPSC-ASPM-2 and hiPSC-ASPM-3, presented normal human karyotypes (Fig. S3A). By sequencing the exons of the three hiPSC-ASPM cell clones, we identified a heterozygous mutation at 9,910 C→T ln exon 25 that introduced a premature stop codon and a homozygous mutation at 7,684 A→G in exon 18 that produced a S2562G missense mutation, which is consistent with previously reported *Aspm* mutations in primary microcephaly patients (Bond et al., 2003) (Fig. S3B). All three hiPSC-ASPM cell lines exhibited similar expression levels of the *Aspm* gene, which was down-regulated compared to the hiPSC-control cell clone (Fig. S3C). In addition, we found some pluripotent stem cell marker genes were slightly down regulated in hiPSC-ASPM cell clones (Fig. S3D) and iPSC grew normally (Fig. S3E), suggesting that *Aspm* mutations may not directly change the differentiation potentials in iPSCs. Under our 3D culture system, we observed that all hiPSC-ASPM cell lines displayed smaller embryonic bodies compared to the control (Figs. 4A, 4B, and S4A). 3D aggregates from hiPSC-ASPM cell lines were smaller, less condensed and less neocortex-like morphology than hiPSC-control (Figs. 4A and S4A). On day 36, the hiPSC-control derived neural progenitors reproducibly formed larger and more continuous polarized neuroepithelial structures with a ventricle-like cavity inside, but hiPSC-ASPM cell lines develop aggregates only occasionally as small rosette-like neuroepithelial regions without the structural cell alignments as in the control culture (Fig. 4C). Moreover, the ASPM mutant 3D aggregates exhibited very few cells positive for Pax6, Sox2 and ZO-1 (Figs. 4C and S4B) and displayed fewer Ki67^+^ and pH3^+^ cells (Figs. 4C, 4D, S5A, and S5B). Moreover, the distribution of these cells was discrete and disorganized in all hiPSC-ASPM organoids compared to the control (Figs. 4C and S4B). These overall smaller neural tissues were suggestive of the reduced brain size observed in patients (Bond et al., 2002).

To test the proliferation defect, we independently performed RNAi knockdown of *Aspm* by electroporating GFP expressing shRNA in organoids. The endogenous *Aspm* RNAi efficiency was first verified by RT-qPCR (Fig. S5C). Knockdown of *Aspm* in hiPSC-control organoids led to a decrease of Ki67^+^ and pH3^+^ cells close to ventricle (Fig. S5D–F), consistent with the defective phenotype observed in ASPM patient organoids. These findings support the conclusion that ASPM mutant could induce deficiency in proliferation of progenitors. These patient-derived cerebral organoids provided a unique opportunity to examine the hypoplasia observed in microcephaly.

Since organoids generated from hiPSC-ASPM-2 exhibited the more condensed cell mass and intact morphology than organoids derived from hiPSC-ASPM-1 or hiPSC-ASPM-3 (Figs. 4A and S4A), and all of organoids showed similar developing phenotypes, we utilized hiPSC-ASPM-2 as a representative for more detailed studies. Consistent with observations at early culture time, organized lumen structures were hardly maintained in mutants on day 65 (Fig. 4E). We could observe dividing Sox2^+^/P-vim^+^ vRG cells along the apical side in hiPSC-control tissues but not hiPSC-ASPM organoids (Fig. 4E). The amount of vRG and oRG cells in hiPSC-ASPM organoids was significantly reduced compared to control (Fig. 4E). On day 65, Tbr2^+^ IPCs primarily localized outside of but close to the VZ, showing a pattern similar to that of the human developing cortex (Fig. 1E). Unsurprisingly, Tbr2^+^ cells were discrete and disorganized in mutant organoids (Fig. 4E). In summary, the loss of lumen structure and neural progenitor cells in hiPSC-ASPM organoids might mimic the pathogenesis of primary microcephaly in human fetal brain development. Although hiPSC-ASPM organoids developed towards telencephalon revealed by FoxG1 staining, the elaborative lamination was not observed in *Aspm* mutant 3D aggregations, suggesting that the culture system was able to model cortical development diseases.

### Calcium imaging data of control and mutant organoids

**Figure 5:**
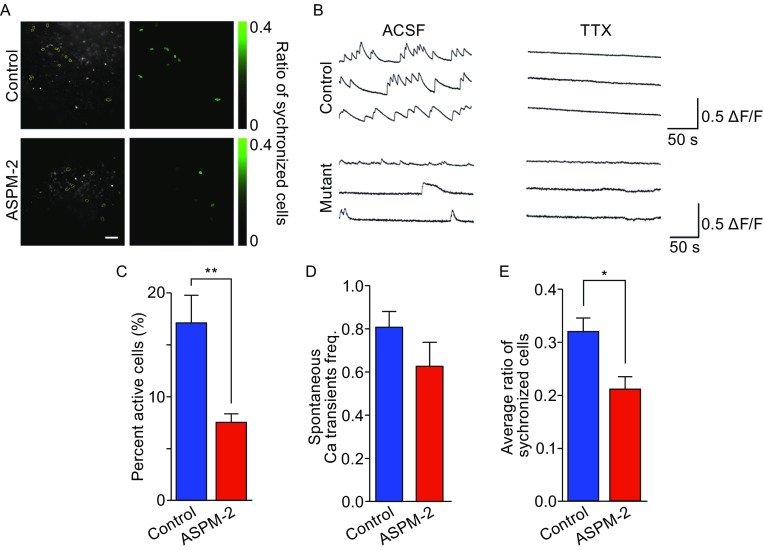
**Calcium imaging results of control and mutant organoids**. (A) Representative images of synchronized calcium activity of cells in control and ASPM-2 organoids. (B) Representative traces before and after TTX-treated in control and mutant organoids. (C) Percentage of active cells in control and ASPM-2 organoids, *n* = 8, 9 trials for control and ASPM-2 groups. (D) Average spontaneous calcium transients of single cells in control and ASPM-2 organoids, *n* = 78, 47 cells for control and ASPM-2 groups. (E) Averaged ratio of synchronized cells in control and ASPM-2 organoids, *n* = 5, 5 trials for control and ASPM-2 groups. All data are presented as means ± s.e.m. *P < 0.05; **P < 0.01. Scale bar: 50 μm in (A)

In hiPSC-ASPM aggregates, although abnormal lamination of neurons was not observed, many Satb2^+^ or Ctip2^+^ neurons were scattered throughout the cell masses (Figs. 4H, 4I, and S4C); therefore, we asked whether these neurons share the same properties as neurons in hiPSC-control organoids. Previous electrophysiological analyses suggested that the neurons began to mature after day 62 (Fig. 3B); hence, we examined calcium activity in organoids on day 85. TTX-sensitive calcium events could be detected in control and mutant organoids (Fig. 5A and 5B), suggesting the presence of active neurons in both hiPSC-control and hiPSC-ASPM-2 aggregates. However, the proportion of cells with calcium activity was reduced in mutants compared to control on day 85 (Fig. 5C), indicating that there were fewer mature neurons in hiPSC-ASPM-2 aggregates than in controls, which may induced by defects in proliferation of progenitors (Figs. 4C, 4D, S5A, and S5B) but not by neuron apoptosis (Fig. S5G and S5H). We analyzed the neurons with active calcium signals and observed that the spontaneous calcium transient frequencies were similar to those of controls (Fig. 5D), but the active cells were not synchronized as well as controls (Fig. 5E), suggesting the neural circuit in hiPSC-ASPM-2 organoids may be dysfunctional. These results indicate that although a less organized structure and fewer mature neurons were observed in hiPSC-ASPM culture, these neurons were able to be activated; however, these neurons were less synchronized and neuronal circuit was not as mature as control group.

## Discussion

In this report, we have established an approach to generate cerebral organoids from hESCs or hiPSCs following dissociation, self-aggregation, and differentiation with minimal extrinsic signals added to the culture system (Fig. 1A). This three-dimensional culture method recapitulates *in vivo* cortical development with formation of a well-polarized ventricle neuroepithelial structure; vRG, oRG and IP cells; and production of mature neurons within layers (Figs. 1–3).

Cerebral organoid culture has been developed and applied widely in cortical developmental diseases. Primary microcephaly with *CDK5RAP2* mutation and Zika virus infection were studied by cerebral organoid culture suggests that 3D culture could recapitulate real *in vivo* organ development to some extent (Lancaster et al., 2013; Tang et al., 2016). Since this system displays fundamental characteristics of human telencephalon cortical development, it is a promising approach to study the pathogenesis of neurodevelopmental disorders. Here, we have modeled microcephaly by generating organoids from the iPSCs of ASPM-mutation patients. Mutations in the *Aspm* gene at the *MCPH5* locus are the most common cause of human primary microcephaly leading to a reduction of cerebral cortex size, congenital failure and mental retardation (Bond et al., 2002, 2003). We have observed defects of early development in aggregates from hiPSC-ASPM cells characterized by a less organized neuroepithelium and fewer vRG, oRG, and defective layer lamination (Figs. 4 and S4). Studies in *Drosophila* suggest that ASPM may interact with myosin II in regulating spindle orientation and interkinetic nuclear migration (INM) in neural progenitor cells (Rujano et al., 2013), suggesting that vRG cells with *Aspm* mutation possibly experienced defective INM, resulting in reduction of oRG cells in the late developmental stages in our culture system (Fig. 4E). This observation could explain why no sufficient ASPM-deletion mouse models that recapitulate microcephaly phenotypes have been developed. Because oRG cells are considered to play a major role in cortical expansion in primates; however, this cell type is limited in rodents (Wang et al., 2011). Cerebral cortical development is an elaborative temporal-spatial progress. Using calcium imaging, we found that fewer neurons had matured and less synchronized neuronal activities had been detected at the late stage of development in the *Aspm* mutants (Fig. 5), which could be the reason that ASPM-related microcephaly patients present congenital failure and mental retardation (Bond et al., 2002, 2003; Shen et al., 2005).

The availability of our novel culture system offers a resource for pathological studies of neurodevelopmental diseases, furthermore, this hESC/hiPSC-derived organoid culture may also be used for various future biomedical applications, including cell therapies, drug discovery and toxicology studies.

## Materials and Methods

### Human induced pluripotent stem cell culture

Cell maintenance and 3D culture procedure are described in SI Materials and Methods.

### Karyotyping and RT-PCR

Karyotypes were determined with standard G-banding chromosome analysis in the Oakland Children Hospital. RT-PCR procedure and primer sequences are in SI Materials and Methods.

### Immunostaining

Cell aggregates were fixed in cold 4% PFA for 30 min at 4°C followed by incubated in 15% (*w*/*v*) sucrose solution overnight at 4°C and then embedded in O.C.T. compound, frozen at −20°C and cryosectioned at 10 μm. Immunostaining and antibody information is available in SI Materials and Methods.

### Flow cytometry

Cell aggregates were fixed in cold 70% alcohol for 30 min at 4°C overnight followed by two washes with PBS. Aggregates were incubated in PBS including Propidium Iodide (PI, 50 μg/mL) and RNase A (50 μg/mL) for 30 min at 4°C. Signals were detected with a FACS Calibur flow cytometer (BD Biosciences). ModFit was used to analysis cell cycle.

### Electrophysiology and calcium imaging

Detail materials and methods are available in SI Materials and Methods.

### Statistical analysis

All data are presented as mean values and standard error of mean (s.e.m.), and two-tailed Student’s *t*-tests were used to determine statistical significance. Cell numbers were counted with Imaris (Bitplane) software and ImageJ. Detail quantification methods are available in SI Materials and Methods.

## Electronic supplementary material

Below is the link to the electronic supplementary material.

Supplementary material 1 (PDF 18249 kb)
