# Supplementary material for: Recapitulating cortical development with organoid culture in vitro and modeling abnormal spindle-like (ASPM related primary) microcephaly disease
Source: Protein Cell. 2017 Oct 23;8(11):823–33. doi: 10.1007/s13238-017-0479-2 (PMC5676597; doi:10.1007/s13238-017-0479-2)
Supplement: Supplementary file 1 — Supplementary material 1 (PDF 18249 kb) [file 13238_2017_479_MOESM1_ESM.pdf]

Supplemental Information

Figure Legends

Fig. S1. Cellular activity of progenitor cells in organoids. (*A*) Representative examples of a double-label staining of P-vim (green), Sox2 (red) demonstrating vRG and oRG cells in different proliferative zone. The percentage of bipolar and unipolar cells that located in VZ/SVZ area respectively. A serious of mitotic vRG cells at different dividing stages in apical surface. (*B*) An example of the mitotic vRG cell ($pH3^+$) division orientation in ventricular surface. (*C*) Quantification of the vRG cell division orientation displayed in bin of 0-30° (horizontal), 30-60°(oblique) and 60-90° (vertical), n=30, anaphase cells from 3 cerebral cortical tissues. (*D*) Representative images of interkenetic nucleus movement of vRG. All data are presented as means ± s.e.m.. Scale bars: 50 μm in (*A*) and 10 μm in (*B*).

Fig. S2. Layer structure during development of cerebral cortex *in vivo*. (*A*) VZ, iSVZ and oSVZ region in human embryonic cerebral cortex revealed by Sox2 and Tbr2 staining. (*B*) Staining for human embryonic cerebral cortex at GW14 labeled with Ctip2 (green), Satb2 (red), Pax6 (blue). Scale bar, 50 μm in (*A*, *B*) .

Fig. S3. The genetic mutations of hiPSC-ASPM. (*A*) Karyotype analysis of the hiPSC-ASPM cell lines. (*B*) Sequencing chromatograms displayed different site mutations on Exons 18 and 25 inherited from three hiPSC-ASPM cell lines. (*C*) RT-PCR (top) and quantitative realtime PCR (bottom) show the expression of *Aspm* genes in hiPSC-control and hiPSC-ASPM cell lines. (*D*) Characterization of iPSC

cell Lines. Relative expression levels of pluripotent stem cell markers by qPCR among hESC (H3 cell line), hiPSC-control, hiPSC-ASPM cell lines and HUVEC. (*E*) Bright field clone images and FACS results of each iPSC lines. All data are presented as means ± s.e.m.. ** *P* <0.01, *** *P* <0.001.

Fig. S4. ASPM patient iPSC-derived neocortex-like organoids display grossly abnormal organization and neuronal differentiation. (*A*) Representative bright field (BF) images of control- and ASPM1, ASPM3-derived organoids at day 1, day 7, day 36. (*B*) Staining for RG progenitors in cortical NE-like areas of the organoids at day 36 derived from hiPSC-control and hiPSC-ASPM1, hiPSC-ASPM3 cell lines, respectively. Sox2 (green), Pax6 (red), ZO-1 (magenta). (*C*) Early-stage cortical neuron differentiation in hiPSC-derived aggregates. Immunostaining of day 36 organoids with Ctip2 (green), Sox2 (red) and DAPI (blue) is shown. Scale bars: 200 μm in (*A*), and 50 μm in (*B* and *C*).

Fig. S5. Apoptosis and proliferation in organoids. (*A* and *B*) Staining results of proliferation marker, Ki67 and pH3, in organoids derived from control, ASPM-1, ASPM-3 iPSC lines, and statistical results, respectively. (*C*) Quantitative realtime PCR result shows the knock down efficiency of *Aspm*-shRNA. (*D* and *E*) hiPSC-control organoids on day 36 electroporated with control or *Aspm*-shRNA plasmid and stained with Ki67 (red) and pH3 (red) after 4 days. High magnification of areas in boxed areas were shown on the right, arrow heads indicate double positive cells and arrows indicate GFP^+ only cells. (*F*) The percentage of $Ki67^+$ or $pH3^+$ and

GFP^+ cells of total GFP^+ cells in control and *Aspm*-shRNA electroporated organoids. (*G* and *H*) Staining results of Caspase3 as apoptosis marker in control and mutant organoids on 65 days, respectively (*G*) and statistical results (*H*). All data are presented as means ± s.e.m.. * $P < 0.05$, ** $P < 0.01$, *** $P < 0.001$, **** $P < 0.0001$. Scale bars: 50 μm in (*A*, *D*, *E* and *G*) and 20μm in (*D* and *E*, right panel).

Supplemental Movies

Movie S1. Radial glial cell division in the sections of organoids derived from the hiPSC-control cells. Radial glia cells were labeled by AAV-GFP virus infection. The normal interkinetic nuclear migration (INM) and cell division of radial glial cells is shown. Images were acquired every 15 min. This movie corresponds to Figure S1*D*.

Materials and Methods

3-D culture and differentiation procedure. Human hiPSCs were maintained on Matrigel-coated 6-well plates (Corning) and were cultured with Essential 8™ Medium (Gibco). On day 0, hiPSCs colonies were pretreated for one hour with 20 μM Y27632 (Tocris Bioscience), and were dissociated to single cells in Accutase (STEMCELL Technologies). Approximately 3×10^6 dissociated hiPSCs in a mixture of KSR medium was plated in AggreWell™ 800 plate (STEMCELL Technologies). The size of EBs is determined by seeding each well of the plate with the total cells mixture. The KSR medium was prepared as follows: DMEM/F12 (Gibco) was supplemented with 20% Knockout Serum Replacement (KSR, Gibco), 2 mM Glutamax (Gibco), 0.1mM nonessential amino acids (NEAA, Gibco), 0.1 mM

beta-mercaptoethanol (Gibco), 3 μM endo-IWR1 (Tocris Bioscience), 0.1 μM LDN-193189 (STEMGENT) and 10 μM SB431542 (Tocris Bioscience). On day 7, the self-organized floating EBs were transferred to low-cell-adhesion 6-well plates (Corning) and further cultured in the neural induction medium containing DMEM/F12, 1:100 N2 supplement (Gibco), 2 mM Glutamax (Gibco), 0.1 mM nonessential amino acids (NEAA, Gibco), 55 μM beta-mercaptoethanol (Gibco), 1 μg/ml heparin. On day 25, the neural induction medium was supplemented with 1% matrigel. After day 50, the free-floating aggregates were transferred to the neurobasal-type differentiation medium supplemented with 1:200 N2，1:50 B27 (Gibco)，2 mM Glutamax and 0.1 mM NEAA. 55 μM beta-mercaptoethanol, 5 μg/ml heparin, 1% matrigel, 10 ng/ml BDNF, 10 ng/ml GDNF, 1 μM cAMP (Sigma).

RT-PCR. Total RNA samples were extracted from four groups of iPSC cell lines using Trizol reagent (Invitrogen). First-strand cDNA synthesis was produced from the total RNA template (1 μg) using a PrimeScript^{TM} 1st Strand cDNA Synthesis Kit (TaKaRa). The cDNA was amplified with a Bio-Rad CFX96 Real-time PCR Detection System using SYBR premix, Ex TaqTM Kit (TaKaRa) and PAGE-purified primers. The PCR conditions were as follows: 40 cycles were run at 94°C for 5 s for denaturation and at 51°C for 30 s for annealing. Primer sequences used for quantitative realtime PCR in the manuscript are *Aspm*(F:5'-GCTAGGCGGTTAA TTGTTC-3';R:5'-AGTTCTCCATAAGTTGTCTCT-3');*Sox2*(F:5'-GGGAAATGGG AGGGGTGCAAAAGAGG-3';R:5'-TTGCGTGAGTGTGGATGGGATTGGTG-3'); *Oct3/4*(F:5'-GACAGGGGGAGGGGAGGAGCTAGG-3';R:5'-CTTCCCTCCAACC

AGTTGCCCCAAAC-3'); *Nanog* (F:5'-CAGCCCCGATTCTTCCACCAGTCCC-3'; R:5'-CGGAAGATTCCCAGTCGGGTTCACC-3');*Fgf4*(F:5'-CTACAACGCCTACG AGTCCTACA-3';R:5'-GTTGCACCAGAAAAGTCAGAGTTG-3');*Gdf3*(F:5'-CTTA TGCTACGTAAAGGAGCTGGG-3';R:5'-GTGCCAACCCAGGTCCCGGAAGTT -3').

Immunostaining. The primary antibodies and dilutions used consisted of the following: anti-phospho-histone H3 (phospho S10) (Abcam, 1:500), anti-Pax6 (Covance, 1:500), anti-FoxG1 (Abcam, 1:1000), anti-Tbr2 (Abcam, 1:300), anti-Sox2 (Santa Cruz, 1:300), anti-phospho-vimentin (MBL International D076-3s (Ser55) or D095-s (Ser82), 1:400), anti-ZO1 (Invitrogen, 1:100), anti-Ctip2 (Abcam, 1:300), anti-Ki67 (Abcam, 1:300), anti-Satb2 (Abcam, 1:200), anti-Brn2 (Santa Cruz, 1:100) anti-Reelin (Millipore, 1:300), . The secondary antibodies included the following: Alexa Fluor 488 (1:1000), 546 (1:1000), and 647 (1:1000) conjugated donkey anti-mouse, anti-rabbit, anti-rat and anti-goat (Invitrogen), Isolectin IB4 Conjugates (Invitrogen, 1:100). DNA was stained with 4', 6-diamidino-2-phenylindole (DAPI, Molecular Probes).

Electrophysiology. Cultured organoids were embedded in 3% low-melting agarose (Fisher) in sucrose-based ACSF (in mM: 234 sucrose, 2.5 KCl, 26 NaHCO3, 1.25 NaH2PO4, 11 D-glucose, 0.5 CaCl2 and 10 MgSO4) and sectioned at 250 or 300 μm in oxygenated (95% O2 and 5% CO2) ice-cold sucrose-based ACSF with a vibratome (VT1200s, Leica). The slices were maintained in a 24-well plate filled with 250 μL of

neural differentiation medium in an incubator (5% CO_2, 37°C). After a recovery period of at least 24 hours, an individual slice was transferred to a recording chamber and continuously superfused with oxygenated ACSF (in mM: 126 NaCl, 3 KCl, 26 $NaHCO_3$, 1.2 NaH_2PO_4, 10 D-glucose, 2.4 $CaCl_2$ and 1.3 $MgCl_2$) at a rate of 3-5 mL per minute at 30±1°C. Whole-cell patch clamp recording was performed on cells from the slices of cultured organoids from day 51 to day 121. Patch pipettes had a 5-7 MΩ resistance when filled with intracellular solution (in mM: 130 potassium gluconate, 16 KCl, 2 $MgCl_2$, 10 HEPES, 0.2 EGTA, 4 Na_2-ATP, 0.4 Na_3-GTP, 0.1% Lucifer Yellow and 0.5% neurobiotin, pH = 7.25, adjusted with KOH). Evoked action potentials were recorded in current-clamp mode using a series of injected currents from -50 pA to 300 pA in increments of 50 pA. Whole-cell currents were recorded in voltage-clamp mode with a basal holding potential of -60 mV followed by stimulating pulses from -80 mV to 40 mV with a step size of 10 mV. The membrane potential was held at -70 mV when spontaneous EPSCs were recorded. The cells were monitored with a 40x Olympus water-immersion objective lens, a microscope (Olympus, BX51 WI) configured for DGC and a camera (Andor iXon3). Stimulus delivery and data acquisition were conducted with a multiclamp 700B amplifier and a Digidata 1440A (Molecular Devices), which were controlled by Clampex 10.

Electroporation. Organoids were injected with 2μl (2μg/μl) GFP expressing plasmids (control and *Aspm*-shRNA) in sterile 1×PBS on 36 day by using a beveled and calibrated micropipette. Then, these organoids were transferred into tweezer electrode (BTX) by a BTX electroporator and shocked with 40 V, 50 ms in duration

with a 950 ms interval for five pulses. After electroporation, these organoids were cultured normally until fixation.

Calcium imaging. The dye solution contained 50 μg Fluo-4 AM (Life Technologies), 50 μL DMSO and 200 μg Pluronic F-127 (Sigma). Then, 3 μl of the dye solution was applied to the surface of each individual slice. After incubation for 30 min at 37°C, the slice was transferred to a recording chamber and continuously superfused with oxygenated ACSF (in mM: 126 NaCl, 3 KCl, 26 NaHCO3, 1.2 NaH2PO4, 10 D-glucose, 2.4 CaCl2 and 1.3 MgCl2) at a rate of 3-5 mL per minute at 30±1°C. The slices were washed 30 minutes before imaging. Calcium imaging was acquired at 5 Hz using a camera (Andor iXon3) with a FITC filter set (Ex: 475/35 nm, Em: 530/43 nm) on a BX51WI microscope (Olympus). Data analysis was performed with ImageJ. The ROIs were selected manually, and the mean fluorescence (F) was calculated for each frame. The fluorescence change over time was calculated as follows: $\Delta F=(F-F_{basal})/F_{background}$, in which F_{basal} was the lowest mean fluorescence value during imaging, and $F_{background}$ was the average mean fluorescence across all frames. Since 10 minutes before imaging, TTX (1 μM) were added by bath application. The slices were rinsed for 30 minutes with ACSF after drug treatments. Calcium transients were identified using a custom-written matlab-based program with a threshold ranged from 0.05 to 0.1 and corrected manually. Averaging ratio of synchronized active cells were calculated by averaging the ratio of synchronized active cells across time.

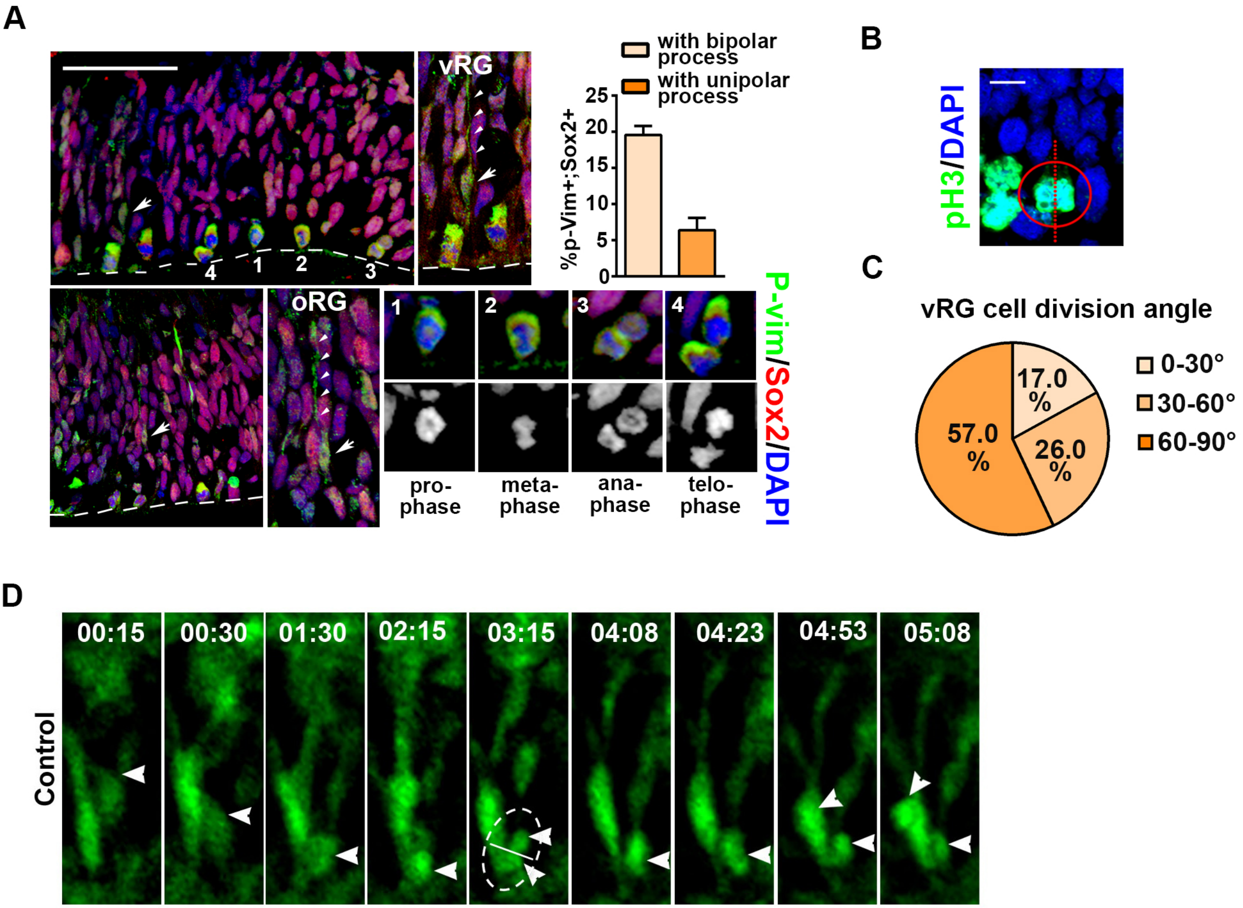
A
vRG
oRG
4
1
2
3
with bipolar process
with unipolar process
%p-Vim+;Sox2+
25
20
15
10
5
0
1
2
3
4
pro-phase
meta-phase
ana-phase
telo-phase
P-vim/Sox2/DAPI
B
pH3/DAPI
C
vRG cell division angle
17.0 %
26.0 %
57.0 %
0-30°
30-60°
60-90°
D
Control
00:15
00:30
01:30
02:15
03:15
04:08
04:23
04:53
05:08

Figure S1

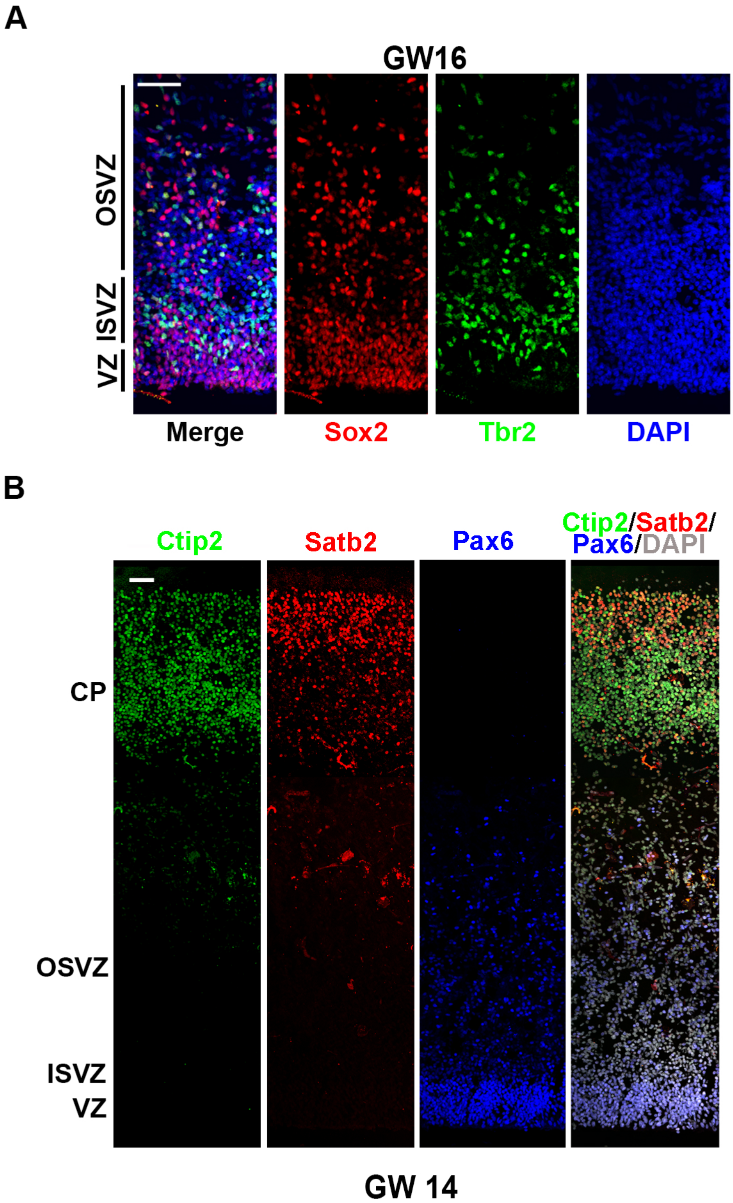
A
GW16
OSVZ
ISVZ
VZ
Merge
Sox2
Tbr2
DAPI
B
Ctip2
Satb2
Pax6
Ctip2/Satb2/Pax6/DAPI
CP
OSVZ
ISVZ
VZ
GW 14

Figure S2

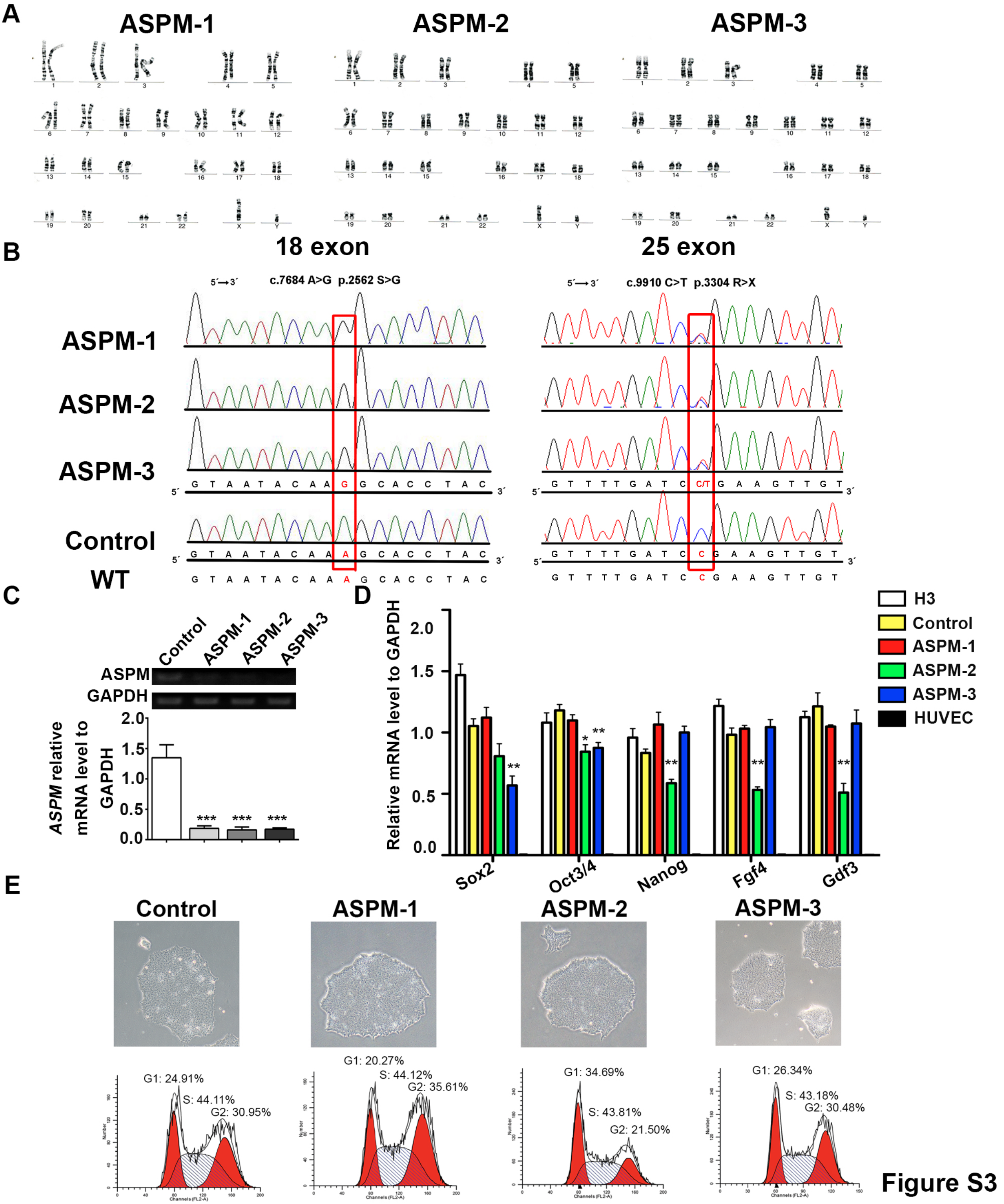
A
ASPM-1
ASPM-2
ASPM-3
B
18 exon
25 exon
c.7684 A>G p.2562 S>G
c.9910 C>T p.3304 R>X
ASPM-1
ASPM-2
ASPM-3
Control
WT
C
Control
ASPM-1
ASPM-2
ASPM-3
ASPM
GAPDH
ASPM relative mRNA level to GAPDH
D
Relative mRNA level to GAPDH
H3
Control
ASPM-1
ASPM-2
ASPM-3
HUVEC
Sox2
Oct3/4
Nanog
Fgf4
Gdf3
E
Control
ASPM-1
ASPM-2
ASPM-3
G1: 24.91%
S: 44.11%
G2: 30.95%
G1: 20.27%
S: 44.12%
G2: 35.61%
G1: 34.69%
S: 43.81%
G2: 21.50%
G1: 26.34%
S: 43.18%
G2: 30.48%

Figure S3

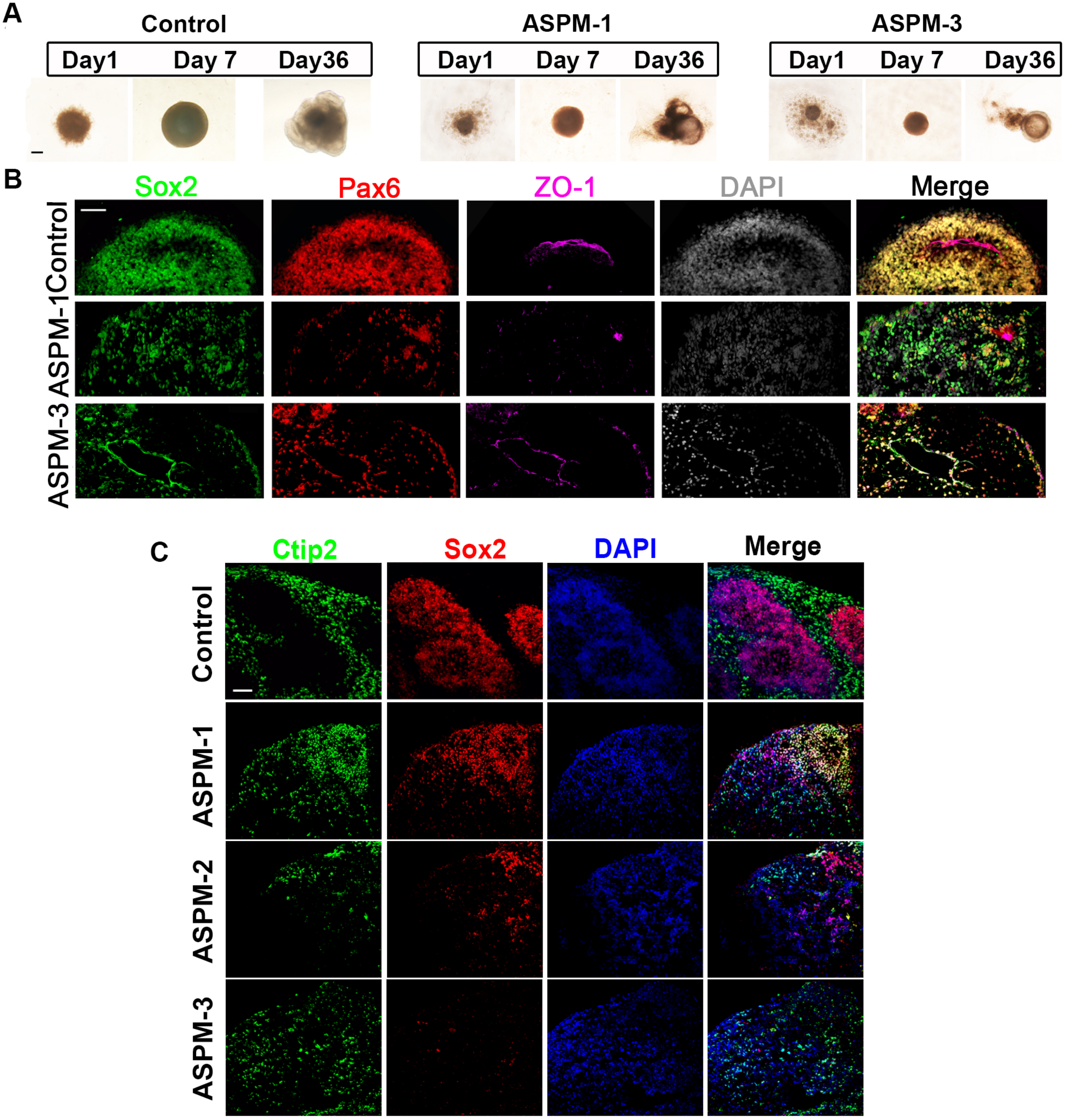

A
Control
Day1
Day 7
Day36
ASPM-1
Day1
Day 7
Day36
ASPM-3
Day1
Day 7
Day36
B
Sox2
Pax6
ZO-1
DAPI
Merge
Control
ASPM-1
ASPM-3
C
Ctip2
Sox2
DAPI
Merge
Control
ASPM-1
ASPM-2
ASPM-3

Figure S4

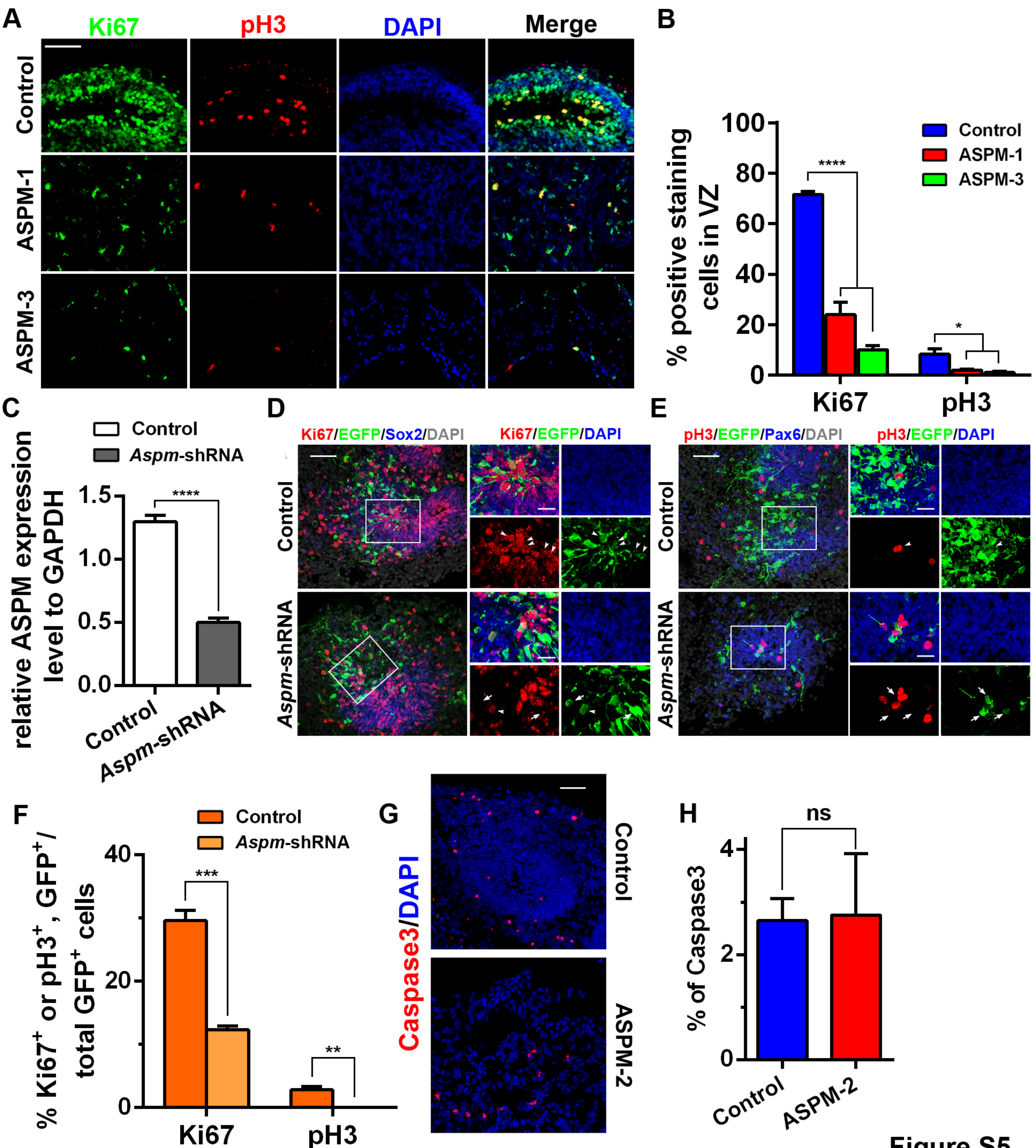

A
Ki67
pH3
DAPI
Merge
Control
ASPM-1
ASPM-3
B
% positive staining cells in VZ
100
80
60
40
20
0
Control
ASPM-1
ASPM-3
****
*
Ki67
pH3
C
Control
Aspm-shRNA
relative ASPM expression level to GAPDH
1.5
1.0
0.5
0.0
****
Control
Aspm-shRNA
D
Ki67/EGFP/Sox2/DAPI
Ki67/EGFP/DAPI
Control
Aspm-shRNA
E
pH3/EGFP/Pax6/DAPI
pH3/EGFP/DAPI
Control
Aspm-shRNA
F
Control
Aspm-shRNA
% Ki67+ or pH3+, GFP+/ total GFP+ cells
40
20
0
***
**
Ki67
pH3
G
Caspase3/DAPI
Control
ASPM-2
H
ns
% of Caspase3
4
2
0
Control
ASPM-2

Figure S5
